# Azygous anterior cerebral artery occlusion managed with Thrombectomy: a case report and literature review

**DOI:** 10.1093/omcr/omaf307

**Published:** 2026-01-28

**Authors:** Muhammad Faisal Wadiwala, Anwar Ibrahim Joudeh, Sheik Akbar Hussein, Mohammed Ibrahim Alhatou

**Affiliations:** Department of Neurology, Al-Khor Hospital, Hamad Medical Corporation, Doha 3050, Qatar; Department of Internal Medicine, Al-Khor Hospital, Hamad Medical Corporation, Doha 3050, Qatar; Department of Medicine, Qatar University, Doha 2713, Qatar; Department of Radiology, Al-Khor Hospital, Hamad Medical Corporation, Doha 3050, Qatar; Department of Neurology, Al-Khor Hospital, Hamad Medical Corporation, Doha 3050, Qatar

**Keywords:** azygous anterior cerebral artery, case report, stroke, Thrombectomy

## Abstract

Azygous anterior cerebral artery (ACA) occlusion is a rare vascular anomaly that results in bilateral frontal infarction and carries a high morbidity rate. We present a 48-year-old male with no prior significant medical history who presented eight hours after sudden onset of right leg weakness. Initially, the National Institutes of Health Stroke Scale (NIHSS) score was four. Computed tomography angiography revealed a solitary azygous ACA with distal A2 occlusion. Mechanical thrombectomy was initially postponed due to mild deficits, but it was performed after clinical deterioration to a NIHSS score of eight. The procedure successfully recanalized the artery. Post-procedure magnetic resonance imaging confirmed bilateral ACA infarcts. The patient received medical therapy and rehabilitation, resulting in complete neurological recovery within three months. This case underscores the diagnostic difficulties associated with azygous ACA occlusion in patients presenting with fluctuating symptoms. Timely observation and intervention were pivotal in preventing severe disability.

## Introduction

Ischemic strokes occurring in the anterior cerebral artery (ACA) territory are relatively uncommon, comprising approximately 0.3% to 4.4% of all ischemic strokes. However, they can result in substantial disability due to the involvement of the medial frontal lobes and corpus callosum [1]. Common clinical manifestations include contralateral leg-predominant weakness, often accompanied by cognitive or behavioral disturbances. Bilateral ACA infarctions are exceptionally rare and are typically associated with anatomical variations, such as the azygous ACA, in which a single pericallosal artery supplies both cerebral hemispheres. The incidence of this vascular variant has been reported to range between 0.3% and 2% [2]. Occlusion of the azygous ACA can lead to catastrophic bifrontal infarctions due to the absence of adequate collateral circulation. Although uncommon, such variants are clinically relevant because they are associated with distinct stroke syndromes and management challenges [2]. Although aneurysms of the azygous ACA are frequently reported, ischemic strokes resulting from its occlusion remain uncommon. Presentations are diverse and may resemble other neurological conditions, while evolving symptoms can further impede recognition [3]. Treatment guidelines for distal ACA occlusions, including the role of mechanical thrombectomy, are poorly defined due to the rarity of the condition.

We present a case of a middle-aged male presenting with progressive bilateral neurological symptoms secondary to azygous ACA occlusion. The patient underwent mechanical thrombectomy and achieved excellent clinical recovery. This report underscores the importance of identifying azygous ACA stroke and contributes to the growing evidence regarding management of this uncommon subtype.

## Case report

**Figure 1:**
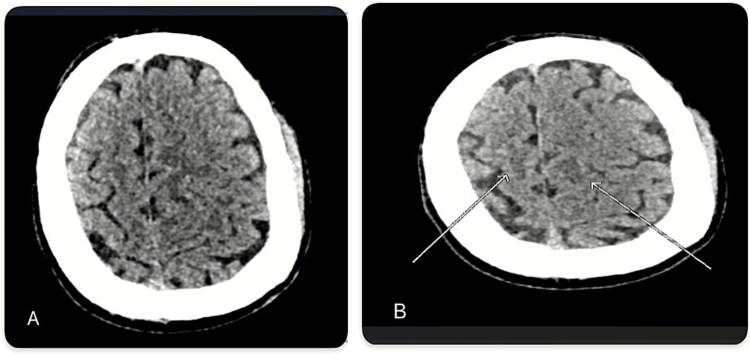
(A and B): Non-contrast computed tomography (CT) imaging demonstrates hypodensity in the left high parasagittal cortex and its underlying white matter, accompanied by faint hypodensity in the right white matter.

**Figure 2:**
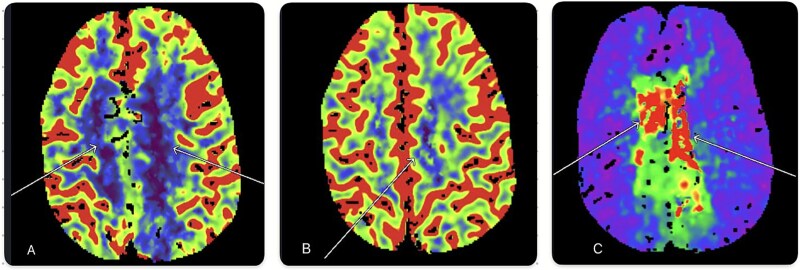
CT perfusion showing (A) reduced cerebral blood flow (CBF) in both anterior cerebral artery (ACA) distributions, (B) partial mismatch in cerebral blood volume (CBV), and (C) prolonged mean transit time (MTT). These findings suggest the presence of an ischemic penumbra.

**Figure 3:**
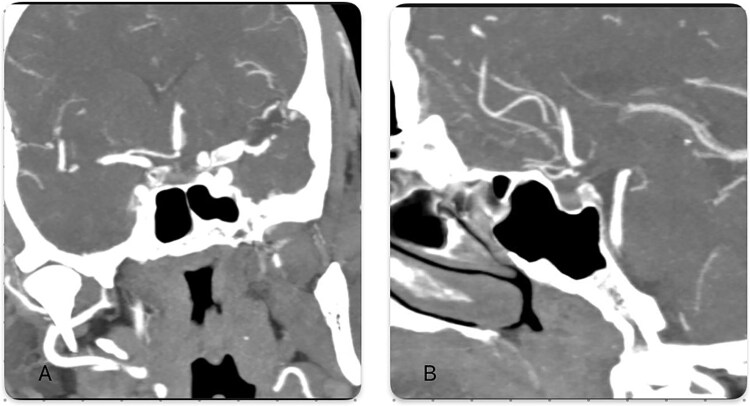
(A) Coronal computed tomography angiography (CT angiography) demonstrates the fusion of the bilateral anterior communicating arteries (A2) into a single midline pericallosal trunk (azygous anterior communicating artery [ACA]). The right anterior communicating artery (A1) is hypoplastic, and the dominant left anterior communicating artery supplies the trunk. A distal cutoff indicates occlusion. (B) Sagittal computed tomography angiography (CTA) reveals the midline ACA branching bilaterally, confirming the azygous configuration. Loss of distal opacification beyond the bifurcation suggests thrombotic occlusion.

A 48-year-old man with no prior medical history presented eight hours after sudden right leg weakness. Neurological examination showed mild right leg weakness and dysarthria (NIHSS 4). Non-contrast head CT revealed no hemorrhage (Fig. 1). CT perfusion demonstrated bilateral high parasagittal parietal hypoperfusion with a visually evident perfusion mismatch consistent with an ischemic penumbra, although no automated quantitative penumbra analysis was available (Fig. 2). CT angiography showed a solitary azygous ACA with abrupt distal A2 occlusion, while other intracranial vessels were patent (Fig. 3). Given that he was outside the intravenous thrombolysis window, had only minor deficits, and the occlusion was situated in a distal ACA branch where the efficacy of thrombectomy is uncertain, the team initially opted for close monitoring rather than immediate intervention.

**Figure 4:**
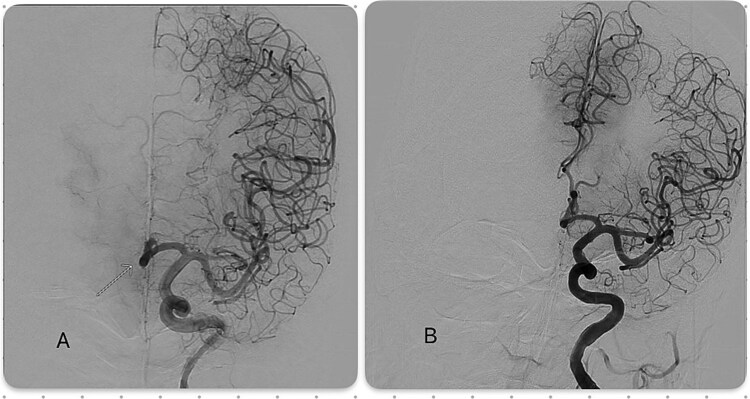
(A) Pre-thrombectomy digital subtraction angiography (DSA) demonstrating an occluded azygous anterior cerebral artery (ACA) with an abrupt distal cutoff. (B) Post-thrombectomy DSA depicting the recanalization of the azygous ACA, resulting in restored distal flow.

Two hours later, his condition worsened (NIHSS 8) with bilateral leg weakness (right > left), right arm drift, dysarthria, and confusion. This rapid neurological worsening, combined with the known azygous ACA anatomy placing both ACA territories at risk, prompted urgent cerebral angiography, which confirmed persistent occlusion. Mechanical thrombectomy using a stent retriever achieved complete recanalization without complications (Fig. 4).

**Figure 5:**
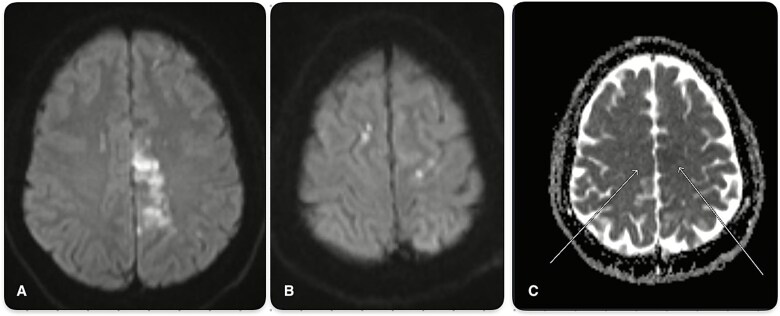
Magnetic resonance imaging (MRI) with diffusion-weighted imaging (DWI b1000) showing (A) acute infraction in the left anterior cerebral artery (ACA) and (B) small foci of acute infarction in bilateral ACA territories. (C) Apparent diffusion coefficient (ADC) scans demonstrate recent infarcts in both ACA territories.

Post-procedure, he was admitted for evaluation and rehabilitation. MRI revealed bilateral ACA territory infarctions involving parasagittal frontal lobes and corpus callosum (Fig. 5). Routine vascular risk screening, including HbA1c, lipid profile, echocardiography, and 48-hour Holter monitoring, was normal. Thrombophilia screening was negative for inherited and acquired causes. Laboratory testing revealed elevated homocysteine (27 μmol/l; normal 0–11 μmol/l) and vitamin B12 deficiency (89 pmol/l; normal 145–596 pmol/l), suggesting a metabolic prothrombotic state likely related to his vegetarian diet.

The patient was treated with aspirin, statin therapy, vitamin B12 supplementation, and intensive rehabilitation. Neurological recovery was rapid, with NIHSS improving to 1 at discharge (day 5). At three months, he demonstrated full recovery (NIHSS 0, modified Rankin Scale [mRS] 0). Persistent hypertension was subsequently detected, and antihypertensive therapy was initiated for secondary prevention.

## Discussion

This case illustrates that azygous ACA occlusion represents a diagnostically challenging and therapeutically demanding stroke subtype. Despite the occlusion being distal, the solitary pericallosal artery supplying both hemispheres caused bilateral neurological deficits and unstable presentations. The lack of collateral circulation in this variant predisposes patients to perfusion instability, necessitating vigilant clinical monitoring and timely reassessment for optimal intervention.

We conducted literature review in the PubMed database for original reports describing azygous ACC-related stroke management using the keywords: azygous anterior cerebral artery AND infarction OR occlusion OR stroke NOT review. We identified 9 cases, of which three cases were excluded because they did not address stroke management, and one case report was identified by cross referencing retrieved articles. A total of seven case reports [3–9] are summarized in Table 1. Most reported patients were for middle-aged to elderly males (50–84 years), with only one female patient. This suggests a male predominance, though the number of cases is small. Presentations were often severe, including akinetic mutism, quadriplegia, aphasia, disturbances of consciousness, tetraparesis, and bilateral frontal infarction syndromes, reflecting azygous ACA unique vascular territory supplying both frontal lobes. Medical therapy alone was the approach in most earlier cases during 2012–2018 [3–6], including antiplatelet therapy, statins, and supportive care. None received IV thrombolysis or thrombectomy. Mechanical thrombectomy, with or without thrombolytics, was reported in later cases [7, 8], demonstrating rapid neurological improvement and favorable outcomes. Medical management alone was associated with unfavorable outcomes, ranging from severe disability (mRS 4–5) to death [3]. Partial recovery was reported in two cases [6, 9], but residual deficits persisted. Thrombectomy cases showed dramatically better outcomes, with NIHSS improvement from 20–32 at presentation to 2 within days to weeks and mRS 0–1 at follow-up, indicating near-complete recovery and independence [7, 8]. Our case aligns with these findings, demonstrating rapid and complete neurological recovery after thrombectomy.

**Table 1:** Summary of case reports on azygous anterior cerebral artery infarction management.

Author (Year) [Reference number]	Age	Sex	Presentation	NIHSS	Management (medical or mechanical thrombectomy)	Outcome
Rajasekharan C (2012) [4]	50	Male	Akinetic mutism/stupor and quadriplegia (acute)	ND	Medical/supportive (no MT reported)	Unfavorable MRS 5 at 3 months (severe disability).
de Sousa CS (2017) [3]	63	Male	Sudden lowering of consciousness; bilateral frontal infarcts	ND	Medical (no IV tPA/no MT reported)	Death (in-hospital).
Khaliq A (2018) [5]	50	Male	Disturbance of consciousness, aphasia, paraparesis/bifrontal infarcts	ND	Medical/supportive (no MT reported)	Unfavorable MRS 4 at discharge.
Saleh C (2018) [6]	62	Female	Headache → somnolence, eye deviation, tetraparesis; seizure noted	ND	Medical (antiplatelet + statin; seizure Rx); aneurysms left untreated	Partial recovery: upper limb strength mostly recovered, persistent spastic paraparesis and cognitive slowing (rehab).
Rangel-Castilla L (2019) [7]	59	Male	Right hemiparesis, facial palsy, aphasia, dysarthria	20	Mechanical thrombectomy (stent retriever + aspiration)	Favourable early outcome NIHSS 2 at 3 days; discharged to rehab.
Asai T (2021) [8]	84	Male	Disturbance of consciousness, quadriparesis, total aphasia	32	IV tPA + Mechanical thrombectomy (contact aspiration)	Favourable NIHSS 2 at 2 weeks; MRS 1 at 90 days (independent).
Ramachandran H (2022) [9]	55	Male	Acute right-sided weakness (predominantly leg) and speech disturbance (left ACA infarct from contralateral carotid stenosis)	ND → improved to NIHSS 9 by 1 month	Medical (dual antiplatelets, statin; planned carotid endarterectomy)	Partial recovery NIHSS 9 and MRS 3 at 1 month; planned carotid endartrectomy

ND: no data available, MRS: Modified Rankin Score, NIHSS: National Institutes of Health Stroke Scale

The present case highlights important considerations. Rare vascular variants like azygous ACA should be considered, especially when patients present with fluctuating or atypical neurological symptoms like paraparesis and abulia. Mechanical thrombectomy may be a valuable treatment option for distal ACA occlusions when initially mild deficits progress. However, this report is limited by its single-case nature, so its generalizability is uncertain. The rarity of published cases of azygous ACA occlusion limits literature and treatment guidelines. Nonetheless, this case supports individualized management and early recognition, timely treatment, and risk factor control to improve outcomes in these rare strokes.
