# Correction to: Azygous anterior cerebral artery occlusion managed with Thrombectomy: a case report and literature review

**DOI:** 10.1093/omcr/omag017

**Published:** 2026-02-18

This is a **correction** to:

Muhammad Faisal Wadiwala, Anwar Ibrahim Joudeh, Akbar Hussein Sheik, Mohammed Ibrahim Alhatou, Azygous anterior cerebral artery occlusion managed with Thrombectomy: a case report and literature review, *Oxford Medical Case Reports*, Volume 2026, Issue 1, January 2026, omaf307, 10.1093/omcr/omaf307

In the originally published version of this manuscript, author, Sheik Akbar Hussein's, forename and surname were inverted

This error has been corrected.

